# Uterine Dendritic Cells Modulation by Mesenchymal Stem Cells Provides A Protective Microenvironment at The Feto-Maternal Interface: Improved Pregnancy Outcome in Abortion-Prone Mice 

**DOI:** 10.22074/cellj.2019.6239

**Published:** 2019-06-15

**Authors:** Maryam Eskandarian, Seyed Mohammad Moazzeni

**Affiliations:** Department of Immunology, Faculty of Medical Sciences, Tarbiat Modares University, Tehran, Iran

**Keywords:** Dendritic Cells, Mesenchymal Stem Cells, Spontaneous Abortion

## Abstract

**Objective:**

Dendritic cells (DCs) as major regulators of the immune response in the decidua play a pivotal role in establishment and maintenance of pregnancy. Immunological disorders are considered to be the main causes of unexplained recurrent spontaneous abortions (RSAs). Recently, we reported that mesenchymal stem cells (MSCs) therapy could improve fetal survival and reduce the abortion rate in abortion-prone mice, although the precise mechanisms of this action are poorly understood. Since MSCs have been shown to exert immunomodulatory effects on the immune cells, especially DCs, this study was performed to investigate the capability of MSCs to modulate the frequency, maturation state, and phenotype of uterine DCs (uDCs) as a potential mechanism for the improvement of pregnancy outcome.

**Materials and Methods:**

In this experimental study, adipose-derived MSCs were intraperitoneally administered to abortion-prone pregnant mice on the fourth day of gestation. On the day 13.5 of pregnancy, after the determination of abortion rates, the frequency, phenotype, and maturation state of uDCs were analyzed using flow cytometry.

**Results:**

Our results indicated that the administration of MSCs, at the implantation window, could significantly decrease the abortion rate and besides, increase the frequency of uDCs. MSCs administration also remarkably decreased the expression of DCs maturation markers (MHC-II, CD86, and CD40) on uDCs. However, we did not find any difference in the expression of CD11b on uDCs in MSCs-treated compared to control mice.

**Conclusion:**

Regarding the mutual role of uDCs in establishment of a particular immunological state required for appropriate implantation, proper maternal immune responses and development of successful pregnancy, it seems that the modulation of uDCs by MSCs could be considered as one of the main mechanisms responsible for the positive effect of MSCs on treatment of RSA.

## Introduction

In allogeneic pregnancy, despite the close contact of the maternal immune system and immunologically foreign fetal-placental alloantigens, the mother’s immune system not only does not reject the fetus but also helps the fetus to implant and develop within the uterus (1). It is well established that in a normal pregnancy, maternal immune responses at the feto-maternal interface are precisely controlled by immunoregulatory mechanisms (1, 2). In contrast, failure in the immune response fine-tuning leads to disturbed pregnancy outcomes such as recurrent spontaneous abortion (RSA) and preeclampsia (2-4). The pattern of the immune cells and immunoregulatory mediators produced within the decidua play a crucial function in the maintenance of tolerance toward the semi-allogeneic fetus (5). Recently, immunological disorders are reported as the main players in the etiology of idiopathic RSA (6).

cells (NK), macrophages (MQ), T lymphocytes, natural killer T cells (NKT), regulatory T cells (Tregs), and Dendritic cells (DCs) are present in the pregnant uteri (7). Among these cells, uterine DCs (uDCs) are considered the major regulators of the immune responses, mainly present at the interface of the innate and acquired immune responses, adjusting T-cell mediated immunity and stimulating the induction of regulatory T-cells, etc. These immunoregulatory mechanisms collectively lead to tolerogenic microenvironment and protection of semiallogeneic embryo (8, 9). uDCs are not only crucial for the generation of maternal immunologic tolerance but also essential for the implantation of embryo via regulating stromal cell differentiation and vascular maturation and remodeling (10). It is supposed that decidual DCs may also play an important role in the etiology of RSA, and any disturbance in their distribution, maturation state, and function could affect the pregnancy outcome that may lead to a disturbed pregnancy (11).

It is well-established that the number, phenotype, and maturation state of DCs determine the tolerogenic or stimulatory nature of the immune response and its intensity (12). uDCs in a normal pregnancy usually have an immature phenotype and are functionally impaired in terms of immunogenic antigen presentation and T-cell activation (12, 13). In contrast, some functional changes in decidual DCs have been reported in pathological conditions such as RSAs and preeclampsia (14, 15). It is well-proven that the tissue environment (including cellular context and secreted factors) profoundly affects the maturity and function of DCs (16, 17). In other words, the behavior of uDCs is extremely controlled by the microenvironment in which they are developed (18). Therefore, it is supposed that the microenvironment of decidua can either foster DCs to promote cell toleration at the fetal-maternal interface or trigger an immune response that is associated with fetal rejection (12).

Regarding the importance of immune system failures, particularly dendritic cells (DCs) malfunctions in unexplained RSA, several therapeutic protocols based on immune modulation have been developed, including paternal leukocyte immunization, and aspirin, progesterone, and immunoglobulins administration. These treatments have yielded some promising results, although several controversial outcomes have also been reported (19). In recent years, the treatment of RSA using MSCs has been implicated due to their immunomodulatory properties, low immunogenicity, and ability to migrate to the site of inflammation prefrentially (20-22).

In our previous study, we showed that the administration of MSCs to an abortion-prone murine model (CBA/J×DBA/2) improved fetal survival and reduced the rate of abortion (20-22). Consequently, we demonstrated that MSCs could be a suitable potential candidate for the treatment of RSA. MSCs have been shown to exert immunomodulatory effects on immune cells, especially DCs. Recent studies have demonstrated a critical role for MSCs in the modulation of DCs differentiation, maturation, and function (23, 24).

In this study, we hypothesized that MSCs might exert their protective effects, at least in part, by modulating the context of the DC of uterine. Thus, the frequency, phenotype, and maturation stage of uDCs in abortion-prone pregnant mice following MSCs therapy were evaluated.

## Materials and Methods

### Mice and mating

In this experimental study, female CBA/J (6-10 weeks old), male BALB/c, and male DBA/2 (8-10 weeks old) mice were obtained from the Pasteur Institute of Iran (Tehran, Iran), housed in an animal facility under optimal condition of temperature, humidity, and 12-hours light/ dark cycle. All animals were handled under procedures approved by the Ethical Committee of Tarbiat Modares University (IR.TMU.REC.1394.286).

Female CBA/J mice were allowed to mate with male DBA/2 or BALB/c mice by overnight cohabitation and then, checked for vaginal plug every morning. The day of vaginal plug observation was considered the day 0.5 of pregnancy.

The mating of female CBA/J mice with male DBA/2 was determined as the immunogenic-abortion mouse model. 1×106 adipose-derived MSCs were intraperitoneally injected to DBA-mated CBA/J females at the implantation period (the day 4.5 of gestation) (MSCs-treated group, n=5). Female CBA/J mice in the control group (CBA/J×DBA/2) just received phosphate-buffered saline (PBS) at the implantation time (n=5). PBS-treated BALB/c-mated females (n=5) were used as the normal pregnant controls.

### Mesenchymal stem cells isolation and characterization

MSCs were obtained from adipose tissue of CBA/J mice (5-7 weeks), as we described previously (20-22). Simply, abdominal fat tissue from non-pregnant CBA/J mice was cut into small pieces and carefully exposed and digested by collagenase type I (Gibco, Germany). The obtained cells were cultured in DMEM supplemented with 10% heatinactivated fetal bovine serum (FBS, Gibco, Germany). Non-adherent hematopoietic cells were removed after 18- 24 hours and adherent cells were cultured to the second passage when the cells were used for the administration. MSCs were characterized through the evaluation of their expressed cell surface markers by flow cytometry (FACSCanto, BD, San Jose, CA, USA) and capability to differentiate into adipocytes and osteoblasts.

### Pregnancy outcome and preparation of uterine cells

Pregnant mice were sacrificed on the day 13.5 of gestation, and their uteri horns were completely removed. The abortion rate was recorded as we described previously (11, 20). After complete removal of fetuses and placenta, the uteri were minced into small fragments and digested using 1mg/ml collagenase IV (Roch, Germany) and 0.2 mg/ml DNase (Sigma-Aldrich, St. Louis, USA). Digested tissue was then filtered through a 70 μm strainer and washed twice in cold PBS. Finally, cells were collected and re-suspended in cold PBS.

### Flow cytometry analysis

**Fig 1:**
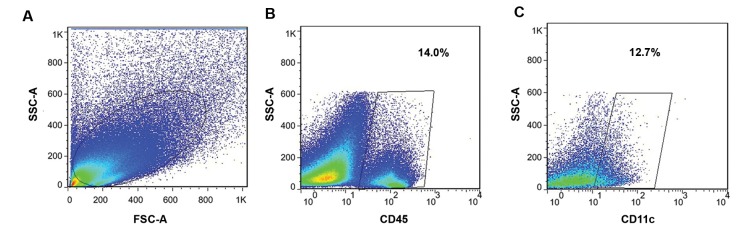
On the day 13.5 of gestation, single cell suspensions were prepared from uteri of MSCs-treated, MSCs-untreated and normal pregnant mice. The cells were stained with monoclonal antibodies against CD45 and CD11c and analyzed by flow cytometry. **A.** Representative dot plots were gated on forward versus side scatter (FSC/SSC) to determine uterine cells population, **B.** CD45^+^ cells were gated on selected uterine cells, and **C.** Than CD11c^+^ cells selected among the CD45^+^ cells to show the percentage of uDCs. MSCs; Mesenchymal stem cells and uDC; Uterine dendritic cells.

Single cells prepared from the uterus were treated with antibody against CD16/CD32 (anti-Fcγ receptor III/ II antibody) to avoid non-specific antibody binding through Fc receptors blockage. Cells were then washed twice with ice-cold PBS (pH=7.2) and stained with PEconjugated hamster anti-mouse CD11c and one of the APC-conjugated monoclonal antibodies (anti-MHCII, anti-CD86, anti-CD11b, and anti-CD40) and APCCy7- conjugated antibody (anti-CD45) (all antibodies obtained from eBioscience, San Diego, USA). Cells were subsequently analyzed by flow cytometry (FACSCanto II, BD, San Jose, CA, USA) and the obtained data were analyzed using the FlowJo software (version 6.07). The uterine cells were selected on dot plots of side and forward scatters. CD45-positive cells as uterine leukocytes were gated and the frequency of CD11c-positive cells (mouse uDCs) was evaluated in uterine leukocyte population (Fig .1). The expression of the DC lineage marker (CD11b) and co-stimulatory molecules (CD40, CD86, and MHCII) were assessed on CD11c^+^ cells.

### Statistical analysis

The differences between the groups were evaluated using a standard parametric test (One- way ANOVA test) followed by Turkey post hoc tests, after approval of the normal distribution of the obtained data by the Kolmogorov-Smirnov test. The results were considered statistically significant if the P was less than 0.05. The results were presented as the mean and standard deviation (mean ± SD) of five separate experiments. All statistical analyses were performed using the Prism software (version 6.07).

## Results

### Characterization of mesenchymal stem cells

Flow cytometry analysis confirmed that MSCs strongly express typical markers such as Sca-1, CD90, CD105, CD29, and CD73 while they were negative for the expression of hematopoietic markers including CD45, CD34, CD11b, and MHC-II. The differentiation potency of MSCs into adipocyte was demonstrated by the observation of triglyceride-containing vacuoles in the cell cytoplasm, by oil red staining. Alizarin-red S staining of calcium accumulation also showed the osteogenic potential of MSCs (data not shown).

### Effect of mesenchymal stem cells therapy on pregnancy outcome

In accordance with our previous results (20, 22) we found that MSCs administration during the implantation window remarkably decreased the abortion rate in the abortion-prone mouse model. A statistically significant lower abortion rate was shown in the MSCs-treated mice compared with the control group (6.5 ± 6.08 vs. 34.6 ± 7.7, P<0.001). The abortion rate in the normal pregnant group was 5.3 ± 4.3.

### Effect of mesenchymal stem cells treatment on uterine dendritic cells

**Fig 2:**
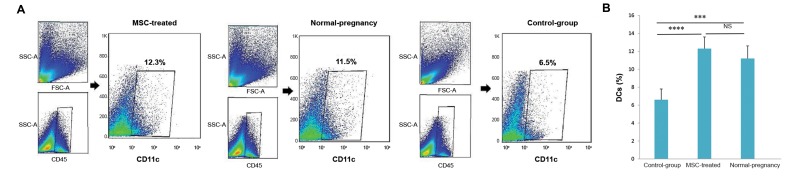
The effect of MSCs administration on frequency of uDCs. A. The dot plots show the percentage of uDCs (CD11c^+^ cells) in MSCs-treated, untreated group (control-group) and normal pregnant groups. The plots are representative of five independent experiment in each group and B. The graph indicates that MSCs administration significantly increased the frequency of DCs in uterine. The differences between the groups were evaluated using a standard parametric test (one-way ANOVA test). The results show the mean ± SD of five independent experiments. ***, ****; p<0.001 and p<0.0001 respectively, MSCs; Mesenchymal stem cells, uDC; Uterine dendritic cells, and NS; Not significant.

The flow cytometry analysis demonstrated that the average density of uDCs was significantly higher in the MSCs-treated mice compared with the control group (12.3 ± 1.5% vs. 6.5 ± 1.3%, P<0.0001, Fig .2). Notably, we observed that the mean frequency of uDCs in the MSCs-treated group was similar to normal pregnant control mice (12.3 ± 1.5% vs. 11.2 ±1.2, P=0.3, Fig .2). Meanwhile, we found that the average percentage of uDCs in the control group (abortion-prone mice) was noticeably lower than the normal pregnant and MSCs-treated groups (6.5 ± 1.3% vs. 11.2 ± 1.2%, P<0.001) and (6.5 ± 1.3% vs. 12.3 ± 1.5%, P<0.0001) respectively (Fig .2).

**Fig 3:**
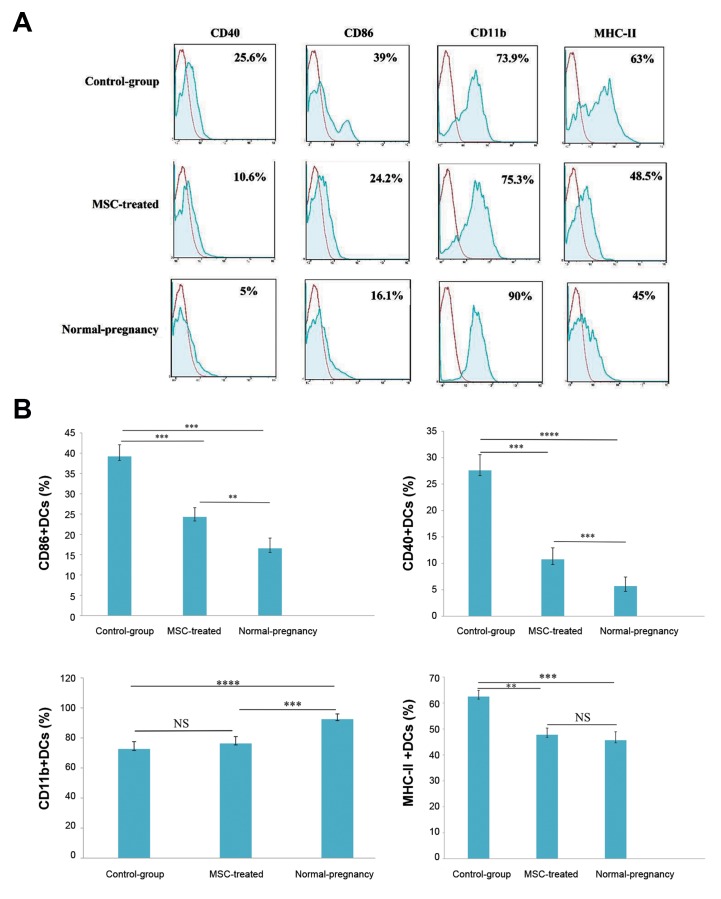
The effect of MSCs administration on the immunophentype of uDCs. The uterine cells were isolated from uterine of MSC-treated, un-treated group (controlgroup) and normal pregnant (normal-pregnancy) mice at the gd 13.5, stained with monoclonal antibodies against CD45, CD11c and one of the monoclonal antibodies (anti-MHC-II, anti-CD86, anti-CD11b and anti-CD40) then analyzed by flow cytometry. A. The CD11c positive uDCs were selected from the CD45^+^ cells of whole uterine cell population. Then the expression of CD11b, CD86, CD40 and MHC-II on uDCs was evaluated .The red histograms show the isotype controls and B. The graphs indicate that MSCs administration significantly decreased the expression of MHC-II and co-stimulatory molecules (CD86, CD40) on uDCs while CD11b^+^ DCs were not changed following MSC therapy. The differences between the groups were evaluated using a standard parametric test (one-way ANOVA test). The results show the mean ± SD of five independent experiments. **, ***, and ****; p<0.01, p<0.001, and p<0.0001 respectively, MSCs; Mesenchymal stem cells, uDC; Uterine dendritic cells, and NS; Not significant.

Further analysis showed that the expression of CD86, CD40, and MHC-II markers on the uDCs in control group (abortion-prone mice) (39.2 ± 2.8%, 27.6 ± 2.9%, and 62.5 ± 2.1% respectively) was noticeably higher than normal pregnant group (16.5 ± 2.5%, 5.7 ± 1.7%, and 45.6 ± 3.2%, respectively, P<0.001, P<0.0001, P<0.001, Fig .3). MSCs administration caused a significant decrease in the expression of the early-mentioned co-stimulatory molecules on uDCs of MSCs-treated mice (24.3 ± 2.2%, 10.7 ± 2.1%, 47.7 ± 2.5%) compared with the control group (P<0.001, P<0.001, P<0.01, Fig .3).

Further investigations indicated that the relative percentage of CD11b^+^ uDCs in normal pregnant mice (92.5 ± 3.5%) was higher than the control group (abortion-prone group) (72.6 ± 4.8%, P<0.0001). Treatment with MSCs did not change the frequency of CD11b-positive cells in uterus compared with the control group (75.3 ± 4.5% vs. 72.6 ± 4.8%, P=0.3, Fig .3).

## Discussion

Because of immunosuppressive properties of MSCs, they display therapeutic efficacy for the treatment of various immune-related diseases such as inflammatory, auto-immune and graft-versus-host (GVH) diseases (23). Many studies have reported that MSCs can diminish the clinical relapse rate in GVHD and ameliorate the function of defective organs in autoimmune disease models (25). Moreover, MSCs transplantation was shown to be safe due to their low immunogenicity (23). However, there are some limitations in the use of stem cells for cell therapy such as the potential malignancy development, finite replicative lifespan, ethical consideration, and the probability of somatic mutation. However, these disadvantages are most common in the case of using embryonic stem cells and induced pluripotent stem cells (iPSCs) not MSCs (26). In our previous studies (20- 22), we also showed that MSCs therapy could improve fetal survival and reduce the abortion rate in abortionprone mice. Many studies have reported that fetal death in this model is related to the aberrant immune response including malfunction of NK cells and MQ, increment of Th1 cytokines, and the reduction of regulatory T cells frequency (27). Understanding the precise mechanisms accounting for the positive effect of MSCs on reducing the abortion rate in abortion-prone mice seems to be crucial. Based on the importance of uDCs in the induction of specific tolerogenic state required for proper maternal immune responses and the establishment of successful pregnancy, we investigated whether MSCs are capable of regulating uDCs recruitment and maturation state during gestation and finally improving pregnancy outcome.

Our results showed that uDCs are significantly less frequent in the uteri of abortion-prone mice compared with the normal pregnant animals. MSCs-therapy caused a significant upregulation in the frequency of uDCs which came close to the normal pregnancy.

It is well-defined that uDCs play a crucial role in the maintenance and development of pregnancy as the activators and regulators of T-cell immunity (12). uDCs are not only essential for the induction of tolerogenic responses against the semi-allogeneic embryo but also play an important role in uterine receptivity and vascular maturation during the implantation of the embryo (10). Fine-balance of uDCs frequency is crucial for the establishment and development of a successful pregnancy. In agreement with this idea, Krey et al. (28) reported that the depletion of uDCs before the implantation leads to pregnancy failure due to disturbed embryo implantation and decidualization. Also, according to Tirado-González et al. (15) the number of decidual DC-SIGN+ cells in human RSA cases were considerably decreased compared with the normal pregnancies. Furthermore, it was shown that the administration of syngeneic DCs to an abortionprone murine model reduces the rate of abortion, yet the mechanism underlying this function is poorly understood (29).

It was shown that MSCs exert immunomodulatory effects on immune cells (especially DCs) through the secretion of various components, as well as a direct cellcell contact (23). Numerous in vitro studies demonstrated that MSCs suppress the generation of myeloid DCs from both monocytes and CD34^+^ cell precursors. However, the immunosuppressive effect of MSCs is related to their surrounding microenvironment, which plays a decisive role in determining their function (30). It is now known that inflammatory cytokines such as IFN-γ and TNF-α augment the immunomodulatory roles of MSCs (20, 31). A large body of research has reported the dominance of inflammatory responses in the decidua, at the beginning of pregnancy and during the implantation period, when we also have administered the MSCs (1). This inflammatory situation not only helps the attraction and migration of MSCs to the uterine but also enhances their immunomodulatory effects (31, 32).

Of note, MSCs produce several cytokines and chemokines, including colony stimulating factor (CSF- 1), granulocyte-monocyte colony stimulating factor (GM-CSF), IL-8, and CCL2, playing major roles in recruiting the immune cells, particularly DCs within the uterus (23). It was shown that GM-CSF could promote DC differentiation in vitro, as well as enhancing DC expansion in vivo (33). MSCs may also regulate the trafficking of immune cells (especially uDCs) toward the endometrium through modulating the secretion of GM-CSF by uterine epithelial cells. Tremellen et al. (34) demonstrated that GM-CSF synthesis is upregulated in uterine epithelial cells by seminal factors, especially TGF-β. TGF-β is also among the most important cytokines secreted by MSCs (23).

Moreover, our findings showed that uDCs in MSCsuntreated abortion-prone mice were more mature compared with the normal pregnant mice. MSCs therapy dramatically decreased the expression of MHC-II and co-stimulatory molecules (CD86, CD40) on uDCs. It is believed that the maturation stage of uDCs also plays an essential role in the etiology of RSA (12). uDCs in normal pregnancy are mostly immature and inefficient for the induction of immunogenic T-cells response (13). Consistent with this idea, Blois et al. proposed that the increased number of mature uDCs might be associated with a high rate of abortion in CBA/J×DBA/2 mating (12). Also, Askelund et al. (35) showed that, at 8 weeks of gestation, mature (CD83^+^) uDCs were significantly more frequent in women with RSA than the normal controls. It seems that these abnormally high immunogenic uDCs can prevail the tolerance to the fetal alloantigens and eventually lead to fetal rejection (12). There is a substantial body of evidence from in vitro studies revealing that MSCs can decrease the expression of MHC-II and co-stimulatory molecules on DCs (23, 36). MSCs secrete critical mediators such as IL-10, TGF-β1, and PGE2, which are major regulators of DCs (23). This immunoregulatory factors prevent the maturation of DCs and induce tolerogenic DCs that are essential for a normal pregnancy (37, 38).

Several studies demonstrated the beneficial effects of stem cell-based therapy on the treatment of inflammatory and autoimmune diseases through the upregulation of anti-inflammatory cytokines, and remarkable reduction in the expression of pro-inflammatory cytokines (39, 40). In our previous studies, we also reported that MSCs therapy in abortion-prone mice could modulate the pattern of inflammatory and anti-inflammatory cytokines (20, 21). Regarding the substantial role of immunoregulatory cytokines (especially IL-10 and TGF-β) in modulating the phenotype and maturation stage of uDCs at the feto-maternal interface (12, 13), it seems that increased release of these cytokines following MSCs-therapy could be taken into account as one of the major mechanisms responsible for the induction of tolerogenic DCs.

## Conclusion

Collectively, our results propose that MSCs therapy can normalize the frequency and maturation state of uDCs in abortion-prone mice. Since, the deregulated immune response is known to be the central player in the etiology of abortion in this model and accepted immunomodulatory effects of MSCs on immune cells especially DCs, as well as considering the key role of uDCs in the induction of tolerogenic response to fetal alloantigens and the development of normal pregnancy, it seems that the modulation of uDCs by MSCs could be one of the primary mechanisms accounting for the positive effect of MSCs in RSA therapy.
